# Quality-by-Design Compounding of Semisolids Using an Electronic Mortar and Pestle Device for Compounding Pharmacies: Uniformity, Stability, and Cleaning

**DOI:** 10.3390/pharmaceutics18020205

**Published:** 2026-02-04

**Authors:** Hudson Polonini, Carolina Schettino Kegele, Savvas Koulouridas, Marcone Augusto Leal de Oliveira

**Affiliations:** 1 Fagron, Fascinatio Boulevard, 350, 3065 WB Rotterdam, The Netherlands; 2 Department of Chemistry, Federal University of Juiz de Fora, Juiz de Fora 36036-900, MG, Brazil

**Keywords:** pharmaceutical compounding, semisolid dosage forms, electronic mortar and pestle, Unguator™, content uniformity, design of experiments (DOE), cleaning validation

## Abstract

**Background/Objectives**: Manual preparation of semisolid formulations (creams, ointments, gels) is prone to variability in mixing energy and time, which may compromise uniform API distribution. This study aimed to evaluate an Electronic Mortar and Pestle (EMP; Unguator™) as a standardized compounding tool, with objectives to: (i) validate stability-indicating UHPLC methods; (ii) assess content uniformity across jar strata; (iii) quantify the impact of mixing time and rotation speed via design of experiments (DOE); and (iv) verify cleaning effectiveness and cross-contamination risk. **Methods**: Five representative formulations were compounded: urea 40%, clobetasol 0.05%, diclofenac 2.5% in hyaluronic acid 3% gel, urea 10% + salicylic acid 1%, and hydroquinone 5%. UHPLC methods were validated per ICH Q2(R2) and stress-tested under acid, base, oxidative, thermal, and UV conditions. Homogeneity was assessed by stratified sampling (top/middle/bottom). A 3^2^ factorial DOE (time: 2/6/10 min; speed: 600/1500/2400 rpm) modeled effects on % label claim and RSD. Cleaning validation employed hydroquinone as a tracer, with swab sampling pre-/post-use and post-sanitization analyzed by HPLC. **Results**: All UHPLC methods met specificity, linearity, precision, accuracy, and sensitivity criteria and were stability-indicating (Rs ≥ 1.5). Formulations achieved 90–110% label claim with strata CV ≤ 5%. DOE revealed speed as the dominant factor for clobetasol, urea, and diclofenac, while time was more influential for salicylic acid; gels exhibited curvature, indicating diminishing returns at high rpm. Model-predicted optima were implementable on the Unguator™ with minor rounding of rpm/time. Cleaning validation confirmed post-sanitization residues below LOQ and <10 ppm acceptance. **Conclusions**: The Unguator™ provides a practical, parameter-controlled route for compounding pharmacies to standardize semisolid preparations, achieving reproducible layer-to-layer content uniformity within predefined criteria under the evaluated conditions through programmable set-points and validated cycles. DOE-derived rpm–time relationships define an operational design space within the studied ranges and support selection of implementable device settings and set-points. Importantly, the DOE-derived “optima” in this study are optimized for assay-based content uniformity (mean % label claim and strata variability). Cleaning validation supports a closed, low-cross-contamination workflow, facilitating consistent routines for both routine and complex formulations. Overall, the work implements selected QbD elements (QTPP—Quality Target Product Profile; CQA—Critical Quality Attribute definition; CPP—Critical Process Parameter identification; operational design space; and a proposed control strategy) and should be viewed as a step toward broader lifecycle QbD implementation in compounding.

## 1. Introduction

Compounded semisolid pharmaceutical preparations (creams, ointments, and gels) require rigorous control of process parameters to ensure quality, uniformity, and stability. In traditional practice, these dosage forms are prepared manually with a mortar and pestle, a technique that, despite long-standing use, exhibits substantial variability and limited reproducibility. The lack of precise control over critical variables (mixing time, shear rate, and applied force) can lead to inconsistent particle dispersion and non-uniform active pharmaceutical ingredient (API) distribution, with implications for therapeutic performance and patient safety. Moreover, manual workflows make it difficult to document a consistent shear history and mixing endpoint across operators and sites, which complicates batch-to-batch comparability and can undermine confidence in assigned beyond-use dates (BUDs) when product-specific stability data are limited [1,2,3].

Demand for standardized, reproducible compounding has increased, driven by quality expectations for extemporaneous preparations and the need to comply with United States Pharmacopeia (USP) and Good Manufacturing Practices (GMP) requirements [4,5]. In the U.S., USP <795> establishes minimum standards for nonsterile compounding, including requirements related to suitable materials and equipment, documentation and control of compounding processes, and the practical assignment of BUDs when stability information is absent or limited [5]. More broadly, compendia/formularies and regulatory frameworks increasingly expect GMP-aligned controls (traceability, defined procedures, and hygiene controls) even where full industrial GMP does not apply [6,7]. Additionally, compendial standards for nonsterile compounding emphasize documented procedures, appropriate equipment, and reproducible processes as foundational controls for quality in extemporaneous preparations [5].

Additionally, automated devices adapted from non-pharmaceutical contexts (e.g., kitchen or industrial mixers) and commonly used by compounding pharmacies generally fail to meet pharmaceutical-grade specifications; in particular, contact materials may not be demonstrated as non-reactive, non-adsorptive, or non-additive as required by USP <795> and GMP, increasing risks of incompatibility or contamination [6,7]. To address these limitations, FagronLab™ developed the Unguator™ specifically for use in compounding pharmacies, as an Electronic Mortar and Pestle (EMP) system (software version 4.8.0-FW-5.2), is purpose-built to support standardized, hygienic, and reproducible semisolid preparation. The device combines a closed mixing environment, programmable control of speed and duration, and patented vertical–horizontal blade motion to promote uniform dispersion and homogenization across semisolid matrices [7]. Pre-validated mixing programs and batch-level parameter recording support standardization and traceability and enable evaluation of selected Quality by Design (QbD)-aligned elements (identification and control of critical process parameters, DOE-informed operating windows, and hygiene controls) by reducing operator dependence and inter-batch variability. Consistent with ICH Q8(R2) pharmaceutical development principles and supported by ICH Q9(R1) quality risk management and ICH Q10 pharmaceutical quality system concepts, the present work applies a QbD-aligned framework to compounding-relevant, measurable quality attributes; however, it does not constitute a full lifecycle QbD program [8,9,10].

Electronic mortar-and-pestle systems have previously been evaluated for dermatological compounding, reporting generally reproducible outcomes for several cream/ointment bases but limitations for some gel systems [6,11]. However, to our knowledge, this is the first systematic performance evaluation of an EMP technology designed for compounding pharmacy practice, covering analytical method validation, forced degradation, content uniformity by stratified sampling, process optimization via design of experiments, and cleaning validation. Five representative formulations were selected to probe distinct physicochemical challenges: (i) urea 40% cream (high solid content), (ii) clobetasol propionate 0.05% cream (low-dose API), (iii) diclofenac sodium 2.5% in 3% hyaluronic acid gel (polymeric matrix), (iv) urea 10% with salicylic acid 1% cream (multicomponent system), and (v) hydroquinone 5% cream (oxidation-sensitive API). The objective was to determine whether the Unguator™ can deliver reproducible, pharmaceutically acceptable semisolid preparations under standardized and practically implementable conditions suitable for routine use in compounding pharmacies. In this work, the DOE-driven optimization endpoint was confined to assay-based content uniformity across jar strata. Importantly, this study was designed as a device-focused performance and QbD evaluation of the Unguator™ EMP; no direct comparison with manual mortar-and-pestle or other mixers was performed.

## 2. Materials and Methods

### 2.1. Materials

APIs and semisolid vehicles (Versatile™ and Nourivan Antiox™) were obtained from Fagron (São Paulo, Brazil). All reagents were of analytical grade and used as received. Water was purified by a Milli-Q^®^ system from Merck Millipore (Burlington, MA, USA). Reference standard stock and working solutions for each API were prepared in accordance with the relevant European Pharmacopoeia monograph recommendations for assay-related preparations and were used for UHPLC calibration and quantification.

The compounding and analytical procedures were conducted using the FagronLab™ Unguator™ Electronic Mortar and Pestle (EMP) system (Scheßlitz, Germany), equipped with programmable speed and time control, closed-system mixing jars (20–500 mL), and standard titanium-nitride-coated blades, and operated with software version 4.8.0-FW-5.2.

### 2.2. QTPP, CQAs, and CPP/CMA Identification (QbD-Aligned Framework)

For clarity, this study was planned using a QbD-aligned structure, consistent with ICH Q8(R2), focused on quality attributes that can be directly measured and controlled in compounding practice [9]. The Quality Target Product Profile (QTPP) for the model semisolid preparations is: (i) topical semisolid in a closed mixing jar and final container; (ii) labeled strength as compounded; (iii) layer-to-layer content uniformity assessed by stratified sampling (acceptance: 90–110% label claim in all layers and strata CV ≤ 5%); (iv) chemical stability over the assigned beyond-use period assessed via stability-indicating assay capability; (v) acceptable physical appearance and usability (no gross agglomeration and no excessive air entrapment); and (vi) minimized cross-contamination risk between batches.

Based on the QTPP, the Critical Quality Attributes (CQAs) explicitly addressed in this work were: (a) assay/content uniformity across jar strata (primary CQA); (b) stability-indicating assay capability and degradation behavior (supporting CQA); and (c) post-cleaning residue/cross-contamination risk (hygiene CQA). Other CQAs relevant to semisolids (e.g., rheology/viscosity, droplet/particle size distribution, pH, microbial quality, and in vitro release) are acknowledged but were not quantified in the present study.

The Critical Process Parameters (CPPs) experimentally evaluated were mixing rotation speed (rpm) and mixing time (min), as defined in the DOE (Section 2.9). Additional candidate CPPs identified through process mapping include incorporation order, scraping/handling steps, jar fill volume/headspace, and selection of pre-validated device programs (Emulsion/Emulsion+). Candidate Critical Material Attributes (CMAs) include API particle size/wettability and density mismatch, solids loading, and vehicle rheology. A condensed FMEA-style CPP/CMA–CQA mapping and risk ranking, together with a proposed control strategy informed by the present data, is provided in Section 4.

### 2.3. Formulation Design

Five representative semisolid formulations were selected to challenge the mixing and homogenization performance of the EMP system (Table 1). The set intentionally spans diverse rheological and physicochemical profiles: high solid content, low-dose API, polymeric gel, multicomponent system, and an oxidation-sensitive API.

### 2.4. Compounding Procedure

All formulations were prepared as 100 g batches using the Unguator™ EMP under ambient laboratory conditions (22 ± 2 °C; 45–55% RH). Unless otherwise specified, raw materials were weighed gravimetrically to ±0.01 g using a Pioneer^TM^ balance from Ohaus (Parsippany, NJ, USA).

#### 2.4.1. Standard Single-API Protocol (F1, F2, F5)

Single-API creams were prepared in the Unguator™ EMP under ambient conditions. For each batch, 50% of the target vehicle mass was first charged into the mixing jar, after which the active pharmaceutical ingredient was added and covered with the remaining vehicle. The jar was then sealed and mixed according to the assigned experimental condition (Section 2.4.3). Upon completion, the mixing blade was removed, the preparation was visually inspected for uniformity and absence of agglomerates, and the product was packaged in opaque white containers.

#### 2.4.2. Multicomponent Cream Protocol (F4)

The multicomponent keratolytic cream was compounded using the same sequence as above to promote progressive wetting and dispersion. The lower-dose component was introduced first, followed by the higher-load component, before bulk homogenization under the designated mixing condition. After mixing, blades were removed, the product was inspected for homogeneity, and it was packaged in opaque white containers.

#### 2.4.3. Mixing Programs and Experimental Factors (All Formulations)

Each formulation was prepared across a predefined matrix of EMP conditions comprising mixing speeds of 600, 1500, and 2400 rpm and mixing times of 2, 6, and 10 min. In addition to these manual settings, the built-in Emulsion and Emulsion+ programs were applied to emulate standardized, GMP-like mixing profiles. All preparations were performed in triplicate (*n* = 3 per condition).

#### 2.4.4. Two-Step Gel Preparation and API Incorporation (F3)

The polymeric gel was produced in two stages. First, the hyaluronic acid gel base was prepared by weighing hyaluronic acid, phenoxyethanol (0.5% *w*/*w*), and purified water to q.s. 100%, transferring the components to a 2 kg Unguator-compatible FagronLab^TM^ jar, and homogenizing using the Gel program until a uniform dispersion was obtained. The gel was then allowed to rest for 24 h at room temperature to complete hydration and reach the target viscosity, after which it was visually inspected and stored in opaque white bottles. In the second stage, diclofenac sodium (at the target strength specified in Table 1) and ethoxydiglycol (10% *w*/*w*) were accurately weighed. Approximately half of the prehydrated gel base was charged into a 100 mL FagronLab^TM^ bottle, the diclofenac and ethoxydiglycol were incorporated, and the remaining gel base was added. The mixture was homogenized in the EMP using the assigned rpm–time setting or preset program outlined in Section 2.4.3. Following mixing, blades were removed, the gel was inspected for uniformity and absence of visible particulates, and the final product was packaged in opaque white bottles.

### 2.5. Chromatographic Analysis

Quantification of APIs was performed using Ultra-High Performance Liquid Chromatography (UHPLC) in a Vanquish Flex System from ThermoFischer Scientific (Basel, Switzerland) with a Chromeleon™ software v. 7.3 (same manufacturer) integrated. Individual chromatographic conditions (mobile phase composition, flow rate, detection wavelength, and column type) were optimized for each API and are summarized in Table 2. The methods were designed to be stability-indicating and validated according to ICH Q2(R2) guidelines [16].

### 2.6. Methods Validation

The analytical methods were validated for specificity, linearity, precision, accuracy, limit of detection (LOD), and limit of quantification (LOQ) as per ICH Q2(R2) recommendations [16].

Specificity was assessed using the solutions described above by performing UHPLC analyses of (i) API standard solution, (ii) vehicle blank, and (iii) mobile phase (diluent) blank. Forced-degradation samples were prepared using the complete formulation matrix (API in the corresponding vehicle/excipient system), rather than API-only solutions, to capture matrix-related interferences and degradation behavior relevant to compounded products. Interference was considered acceptable when no co-eluting peaks were observed at the API retention time and the relative difference in peak area between standard and matrix-added chromatograms was <2.00%. In accordance with ICH Q2(R2) [9] expectations for stability-indicating methods, forced-degradation studies were also performed as part of the specificity assessment under acidic hydrolysis (6 M HCl), basic hydrolysis (10 M NaOH), oxidative conditions (35% H_2_O_2_), thermal stress (70 °C for 24 h), and photolysis (UV light at 365 nm for 24 h); chromatograms were evaluated for adequate separation of degradation peaks from the parent analyte (target Rs ≥ 1.5). Each forced-degradation condition was analyzed in sextuplicate (*n* = 6). Additionally, photolysis was applied as a forced-degradation stress condition (UV 365 nm for 24 h) to support the specificity/stability-indicating assessment; photolysis was applied as a forced-degradation stress condition to support specificity (stability-indicating capability), not as an ICH Q1B-compliant photostability study (no controlled/reportable cumulative light exposure in lux·h and W·h/m^2^). Photolysis-stressed samples were compared to an unstressed control prepared in parallel but not submitted to photolysis (fresh/unexposed sample of the same matrix and concentration), with both samples processed using the same extraction/dilution protocol prior to UHPLC–PDA analysis. Evaluation was based on the appearance of additional peaks and/or change in the parent peak area relative to the unstressed control, together with PDA peak-purity assessment and chromatographic separation. Specificity assessments were performed in triplicate, and forced-degradation samples were analyzed under each stress condition. Peak purity of the parent API peak in forced-degradation samples was assessed using PDA spectral analysis in Chromeleon™ software.

Precision was evaluated in terms of repeatability and intermediate precision. Repeatability was determined by consecutive analysis of six replicate injections of the API solution at working concentration by a single analyst on the same day. Intermediate precision was assessed by two different analysts on two separate days, each performing six replicate analyses under the same experimental conditions. Precision was expressed as the coefficient of variation (CV), with an acceptance criterion of ≤5%.

Accuracy was determined by spiking the matrix with known amounts of the API at the same concentration levels used in the linearity assessment (*n* = 3 for each level). Analyses were performed by the same analyst, and results were expressed as percent recovery relative to the calibration curve obtained from the linearity study.

Linearity was established from three independent calibration curves constructed using the API concentrations described in Table 1. The curves used a minimum of five calibration levels (*n* = 5) spanning the intended quantification range (from approximately 70% to 130% of the target assay concentration for each API). The relationship between analyte concentration and peak area was evaluated using the ordinary least-squares regression (unweighted) method, using peak area versus nominal concentration. The linearity of each calibration curve was assessed by analysis of variance (ANOVA), and correlation coefficients (r^2^) were determined to quantify the strength of the linear relationship.

The limits of detection (LOD) and quantification (LOQ) were determined from three standard calibration curves and calculated according to Equations (1) and (2), respectively:

(1) $$LD=\frac{3.3\times \sigma }{S}$$

(2) $$\;LQ=\frac{10\times \sigma }{S}$$

where S is the mean slope of the analytical curves and σ is the standard deviation of the response (noise) estimated from at least ten blank sample analyses.

Robustness (e.g., deliberate small variations in flow rate, column temperature, mobile-phase composition, or detection wavelength) was not formally assessed in this study.

### 2.7. Homogeneity and Content Uniformity

Homogeneity testing was performed by stratified sampling from the top, middle, and bottom portions of each compounded jar. For each experimental condition, three independent jars were prepared (*n* = 3), and, for each jar, each layer was sampled and analyzed in triplicate using validated UHPLC methods. Acceptance criteria were applied per jar: the mean API content of each layer had to be 90–110% of label claim, and the coefficient of variation (CV) of the three-layer means (top/middle/bottom) had to be ≤5%. These internal criteria were selected to reflect pharmacopeial expectations for assay accuracy and low variability in compounded preparations, noting that pharmacopeias do not prescribe a specific stratified “content-uniformity” test for semisolid jars. This evaluation assessed vertical distribution and mixing efficiency across the EMP’s operating parameters.

### 2.8. Design of Experiments (DOE)

A 3^2^ factorial design was employed to identify mixing conditions that optimize assay-based content uniformity (mean % label claim and variability), with mixing time (X_1_: 2, 6, 10 min) and rotation speed (X_2_: 600, 1500, 2400 rpm) as independent variables (Table 3). These two critical process parameters were selected based on prior knowledge of their direct influence on the homogenization efficiency of semisolid formulations. The DOE was intentionally restricted to these two factors to maintain feasibility across five formulation matrices (9 conditions × triplicate jars per condition) and to isolate the primary, directly programmable energy-input variables of the EMP. Other potentially influential parameters (e.g., jar size/fill level and headspace, blade configuration/orientation, initial product temperature, and staging of component addition) were standardized. DoE model calculations and regression analyses were performed in Microsoft Excel (Microsoft Corporation, Redmond, WA, USA; version 2510), and response surfaces were generated from the fitted quadratic equations.

The design enabled simultaneous investigation of main effects and second-order interactions on the response variables, defined as (i) API content uniformity (%) and (ii) relative standard deviation (RSD, %). The experiments were conducted in randomized order to minimize systematic error, and each combination was performed in triplicate for each formulation type (*n* = 9 per DOE matrix). The DOE approach facilitated the construction of quadratic polynomial response surface models, allowing prediction and optimization of the most suitable process parameters for each formulation matrix. Accordingly, the predicted ‘optima’ reported from the response-surface models should be interpreted as optima for content uniformity outcomes only.

#### 2.8.1. Phase 1—Performance Analysis

In the first phase, each formulation described in Section 2.2 was analyzed in triplicate to assess intra-batch homogeneity. Samples were collected from the top, middle, and bottom layers of the compounded preparations to evaluate vertical uniformity. The API content in each section was determined using the validated UHPLC methods described previously. The acceptance criteria were defined as an API content between 90–110% of label claim and a coefficient of variation ≤ 5% among layers. The ≤5% CV threshold across the three-layer means (top/middle/bottom) was defined as an internal, fit-for-purpose homogeneity target to indicate minimal vertical stratification in compounded semisolids, recognizing that pharmacopeias do not prescribe a specific stratified ‘content-uniformity’ test for jarred semisolids. This value was selected as a conservative benchmark consistent with typical expectations for low assay variability in quantitative methods and provides a practical, stringent criterion for standardized mixing performance in heterogeneous semisolid matrices.

#### 2.8.2. Phase 2—Response Surface Evaluation

In the second phase, the factorial design was applied to model and optimize the relationship between the process parameters (mixing time and rotation speed) and the resulting homogeneity of the formulations.

The coded levels (−1, 0, +1) represented the low, central, and high values of each factor, respectively. A total of 90 experimental runs were performed across all formulations, including 10 replicates at the central point to estimate experimental error and assess model reproducibility (Table 3).

Experimental data were used to fit a second-order polynomial equation of the form:

Y = β_0_ + β_1_X_1_ + β_2_X_2_ + β_12_X_1_X_2_ + β_11_X_12_ + β_22_X_22_ + ε

where Y is the predicted response (content uniformity or RSD), β_0_ is the intercept, β_1_–β_22_ are the regression coefficients, and ε is the residual error term.

The resulting response surfaces and contour plots were generated to visualize the effects and interactions of the independent variables on the measured responses. The optimal mixing conditions were defined based on the criteria of maximum homogeneity (highest API uniformity, lowest RSD) while maintaining practical compounding time and operational feasibility.

### 2.9. Cleaning and Cross-Contamination Validation

The workbench, equipment, and mixing blades were cleaned following laboratory hygiene standards. The workbench was sanitized with a 2.2% sodium hypochlorite solution, applied using a squeeze bottle and spread with paper towels in unidirectional, uniform movements. The surface was left to air dry before use. The equipment was cleaned using a non-woven cloth moistened with 70% alcohol, ensuring proper disinfection without the use of sharp tools or abrasive agents to avoid damage. Mixing blades were cleaned with a non-woven cloth, rinsed under hot running water, and dried with the same cloth.

To assess environmental and cross-contamination risks after completion of Phase 2, the formulation with the lowest detection limit, hydroquinone, was selected as a tracer compound. Its high analytical sensitivity allowed the detection of even trace residues, ensuring accurate evaluation of potential contamination. Swab samples were collected from three critical surfaces—the workbench, the mixing paddle, and the external housing of the equipment—at three time points: before compounding, after compounding, and after sanitization. Swab sampling was performed using sterile Olen^®^ swabs with plastic shafts and cotton tips (ethylene oxide sterilized, individually packaged). Samples were collected from three critical surfaces: the workbench (40 cm^2^ area surrounding the equipment), the mixing paddle (6 cm of the blade), and the paddle insertion point (7 cm). Each swab was pre-moistened with the hydroquinone diluent solution and applied using unidirectional, uniform strokes with firm pressure while rotating the swab to maximize contact. Following sampling, each swab was placed into 5 mL of hydroquinone diluent solution for extraction, and the resulting extract solutions were analyzed by UHPLC using the validated method. An acceptance criterion of <10 ppm hydroquinone in the 5 mL swab extract (≤50 µg recovered per swab) was adopted as a pragmatic, conservative proof-of-concept threshold for routine compounding hygiene; toxicology-derived Health-Based Exposure Limit/Permitted Daily Exposure (HBEL/PDE) limits and full ‘worst-case’ bracketing across APIs/vehicles were outside the scope of this study.

## 3. Results

### 3.1. Analytical Method Validation

All analytical methods developed for quantifying the active pharmaceutical ingredients (APIs) demonstrated adequate performance according to ICH Q2(R2) criteria (Table 4). Acceptance criteria were defined a priori in an internal analytical-validation SOP, following the fitness-for-purpose principle and the expectation that laboratories predefine method performance criteria prior to validation. For the intended use (assay of APIs in compounded semisolid matrices), the following criteria were applied: linearity r ≥ 0.99; significant regression by ANOVA (*p* < 0.05; F_reg_ > F_crit_); specificity as discrepancy < 2.0%; repeatability and intermediate precision CV ≤ 5.0%; and accuracy (recovery) 100% ± 2%. The predefined acceptance criteria are shown in Table 4 together with the corresponding validation results [17,18,19].

The chromatographic methods showed high specificity, with no interference from vehicle or excipient peaks at the analyte retention times. The relative difference in peak areas between standard and matrix-added samples was consistently below 2%, confirming the absence of co-eluting components. Specificity (stability-indicating capability) was further supported through forced-degradation studies under acidic, basic, oxidative, photolytic, and thermal conditions, which generated degradation peaks that were adequately separated from the parent analyte (Rs ≥ 1.5; Table 5). Hydroquinone and clobetasol propionate showed the greatest susceptibility to oxidation and photolysis, respectively, whereas diclofenac sodium and urea were chemically stable under the tested conditions, confirming that each API can be specifically quantified in the presence of degradation products. Because photolysis exposure was not calibrated to ICH Q1B requirements, photolysis results are interpreted only as qualitative forced-stress outcomes supporting method specificity. PDA peak-purity outcomes are summarized as Pass/Flag in Table 5, with the corresponding numeric peak-purity match-factor outputs provided in Supplementary Table S1. Flag outcomes observed under extreme stress were interpreted conservatively in conjunction with the forced-degradation chromatograms, which still demonstrated separation of degradant peaks from the parent analyte (Rs ≥ 1.5), supporting stability-indicating specificity for assay.

Linearity was established over the tested concentration ranges, with correlation coefficients (R^2^) exceeding 0.999 for all APIs. Precision was confirmed by repeatability and intermediate precision assessments, yielding CV values below 5%, while accuracy results ranged from 98.2 to 101.4% recovery across concentration levels.

The calculated LOD and LOQ values confirmed the high sensitivity of the methods, enabling reliable detection of even minimal residual concentrations, particularly for Hydroquinone. Overall, these results validated that all analytical methods were specific, precise, accurate, and sensitive, ensuring robustness for subsequent performance and contamination assessments.

Formal robustness testing by deliberate variation of method parameters was not performed in this study; consistent with ICH Q2(R2)/ICH Q14 [17,18,19], robustness is typically established during analytical procedure development, and system suitability plus intermediate precision provide operational assurance for the intended use reported here.

### 3.2. Content Uniformity (Homogeneity)

Across formulations, mean recoveries fell within 90–110% and strata CVs were generally ≤5%, indicating effective vertical mixing under the evaluated conditions (Table 6). Preparations containing urea (F1, F4-urea) and hydroquinone (F5) showed tight dispersion (low SD and CV), consistent with efficient dispersion of either high-solid content (urea) or dissolved APIs (hydroquinone) in the tested vehicles. The diclofenac in hyaluronic acid gel (F3) maintained recoveries near 100% with low variability, suggesting adequate API distribution within the polymeric matrix.

By contrast, clobetasol 0.05% (F2) exhibited higher between-strata variability in some runs, with the bottom layer occasionally trending away from the other strata, consistent with partial sedimentation at very low dose levels. In these cases, settings employing longer mixing times and/or higher RPM (or the device’s Emulsion/Emulsion+ programs where applicable) reduced variability, supporting the DOE findings that increased process energy improves dispersion for low-dose suspensions.

Overall, the results demonstrate that the electronic mortar and pestle achieved uniform API distribution throughout the jar for the majority of formulations and settings, and that parameter optimization (time/RPM or program selection) is particularly relevant for low-dose, poorly wetting, or higher-density APIs to fully meet the ≤5% CV target across strata.

### 3.3. DOE Optimization of Mixing Parameters

DoE models were fitted using a full quadratic response surface (coded factors: X_1_ = time; X_2_ = rpm), including linear terms, the interaction (X_1_X_2_), and curvature (X_1_^2^, X_2_^2^). For each formulation, model adequacy was assessed by regression significance (ANOVA) and goodness-of-fit (R^2^). Factor significance was evaluated from the regression/ANOVA outputs (*p*-values), and effect directionality was interpreted from the fitted coefficients and response-surface contours. Full regression outputs, ANOVA tables, and diagnostic plots are available in the Excel DoE files (Data Availability Statement).

Across formulations, factorial models were adequate by ANOVA (significant regression; non-significant lack-of-fit where tested) and residual diagnostics showed no systematic trends, supporting the use of quadratic terms at 3 levels. Time (X_1_) and speed (X_2_) exerted positive main effects on uniformity (higher % label claim convergence and lower RSD), with magnitude depending on formulation rheology and dose. Curvature was present in several matrices (notably viscous or polymeric systems), indicating diminishing returns beyond mid-to-high settings (Table 7).

A modest X_1_ × X_2_ interaction was observed in low-dose emulsions (e.g., clobetasol 0.05%) where simultaneous increases in time and rpm most effectively reduced between-strata variability (mitigating bottom-layer drift noted in Section 3.2). In contrast, polymeric gel (diclofenac in HA 3%) benefited from moderate rpm with adequate time, as excessive speed increased air entrainment risk (manifested as slightly higher RSD without improving mean recovery).

Formulation-specific operating windows (empirical) would be: (i) for low-dose, poorly wetting APIs (F2: clobetasol 0.05%): favor higher rpm (≥1500–2400) and longer time (≥6–10 min); device programs analogous to Emulsion/Emulsion+ reduced RSD across strata; (ii) for high-solid content (F1: urea 40%; F4-urea 10%): mid-to-high rpm with mid time (1500–2400 rpm; ~6 min) achieved tight dispersion; further time gave limited benefit (curvature); (iii) for polymeric gel (F3: diclofenac/HA): moderate rpm (~1500–1900) with sufficient time (≥6 min) balanced shear and bubble control; very high rpm offered no additional uniformity gains (curvature increases); and (iv) for dissolved/antioxidant-protected API (F5: hydroquinone 5%): broad robustness across the design space; mid settings were sufficient to maintain low RSD.

**Figure 1:**
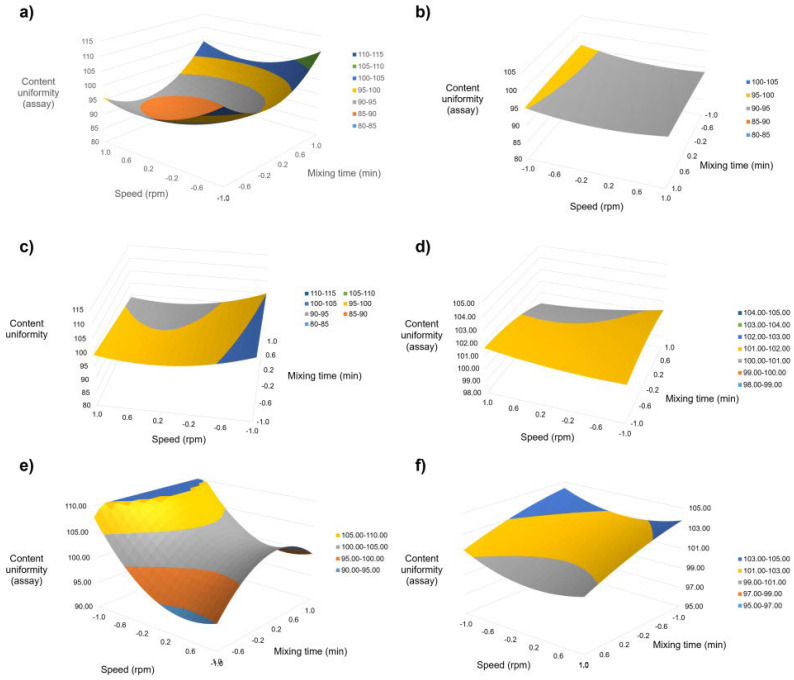
Response surface analysis of mixing time (min) and rotation speed (rpm) on content uniformity (% label claim) of semisolid formulations compounded with the Unguator™ EMP. Dominant main effect of rpm is observed for most matrices; time contributes notably to multicomponent systems. Shaded/contour optima correspond to target criteria (90–110% and minimal RSD). Formulations: (**a**) Clobetasol propionate; (**b**) Urea; (**c**) Diclofenac sodium; (**d**) Urea + Salicylic acid [Urea assay]; (**e**) Urea + Salicylic acid [Salicylic acid assay]; (**f**) Hydroquinone.

Response-surface desirability analysis (maximize uniformity and minimize RSD) identified formulation-specific optima clustered around mid-high rpm with mid-long time (Figure 1). Confirmation runs at the predicted optima reproduced model expectations within experimental error, supporting transferable set-points that can be encoded as standard device programs (and scaled by jar volume) to reduce operator variability.

In practice, the EMP’s programmable control enables recipe-level standardization: (i) select a default window (e.g., 1500–1900 rpm, 6–10 min) for most creams; (ii) elevate energy (≥1900 rpm and/or 10 min) for low-dose suspensions; (iii) cap rpm around 1500–1900 for polymeric gels to limit entrapped air; and (iv) use pre-validated programs (Emulsion/Emulsion+) when thermal steps or multi-phase incorporation are required. These findings mechanistically explain the homogeneity outcomes in Section 3.2 and provide DOE-supported set-points that can be incorporated into a QbD-aligned control strategy for routine compounding.

### 3.4. Translation of DOE Optima to Device Set-Points

To make the design space explicit, Table 8 consolidates the DOE-defined operational design space (within the studied rpm/time ranges) for each formulation, i.e., combinations predicted to meet the homogeneity criterion (90–110% label claim; CV ≤ 5%), meaning that the mixing conditions predicted by the response-surface models to optimize content uniformity were compared with the practically achievable settings on the Unguator™. Predicted model optima and corresponding practical device set-points are summarized in Table 8, and confirmation batches at the practical set-points met the predefined homogeneity criteria.

Minor offsets between model optima and practical set-points reflect the Unguator™’s discrete speed increments and programmed time steps, as well as operational considerations (e.g., air incorporation risk in viscous gels at very high rpm; thermal profiles for Emulsion/Emulsion+). For F2 (low-dose clobetasol), the nearest practicable speed (1650 rpm) paired with 10 min sustained mixing reproduced the uniformity gains predicted at 1860 rpm, consistent with the DOE-identified dominance of process energy. For F3 (diclofenac/HA gel), extending the mixing time at a slightly lower rpm (800 rpm/10 min) achieved the same endpoint while mitigating bubble formation, in line with the curvature observed in viscous systems. F4 demonstrated two viable operating windows; the Unguator™ settings chosen map closely to both modeled optima and support workflow flexibility. F5 (hydroquinone) remained robust across the mid-high region, allowing a small downward adjustment in rpm without loss of content uniformity.

These findings indicate that model-informed set-points can be faithfully implemented on the Unguator™ within its native control scheme, and that equivalent quality outcomes can be achieved through rational trade-offs between time and speed when exact numeric optima are not available. This alignment supports routine deployment of device-encoded recipes for standardized, reproducible compounding.

### 3.5. Cleaning and Cross-Contamination Test

Extracts were analyzed by UHPLC using the validated method; hydroquinone identity was confirmed by retention-time concordance with a reference standard. The acceptance criterion for cleaning validation was <10 ppm (0.001%) hydroquinone. At baseline, no hydroquinone was detected. Immediately after compounding, hydroquinone was detectable but remained below the limit of quantification (LOQ) across sampled surfaces, and in all cases well below 10 ppm. Following the cleaning procedure, no hydroquinone was detected on any surface.

This ≤10 ppm threshold was adopted as a conservative, practical proof-of-concept hygiene criterion for compounding equipment verification and corresponds to ≤50 µg hydroquinone per swab in our 5 mL extraction setup; it was not intended to represent a universal HBEL/PDE-based limit across all APIs and vehicles. Risk-based approaches linking residue limits to dose/toxicology and worst-case bracketing are described in established cleaning-validation guidance documents [18,19,20].

These results demonstrate that, when the prescribed cleaning procedure is applied, the Unguator™ does not retain detectable hydroquinone residues and presents a minimal risk of cross-contamination between batches, supporting compliance with pharmaceutical quality and safety requirements.

## 4. Discussion

The validation and forced-degradation studies confirmed that the UHPLC assays were stability-indicating and suitable for quantifying APIs in semisolid matrices compounded with the Unguator™. Peak purity and baseline resolution of degradants established method specificity, and the predefined performance thresholds for linearity, precision, and accuracy were consistently met. Consequently, subsequent observations on content uniformity and mixing performance can be ascribed to process and formulation factors rather than analytical bias.

Across chemically and rheologically diverse systems, the Unguator™ delivered uniform products for most formulations under the evaluated conditions. Single-API creams with urea and hydroquinone showed tight layer-to-layer agreement and low variability, indicating efficient dispersion in both high-solid and dissolved-API scenarios. By contrast, clobetasol 0.05% and the salicylic-acid component of the urea–salicylic acid combination exhibited greater between-strata variability in some settings, consistent with partial sedimentation or incomplete deagglomeration at low dose or in multicomponent matrices. These observations are coherent with prior reports showing that mixing method and energy input influence microstructure and performance of semisolid systems, including adhesion, rheology, and drug delivery outcomes; bead-mill homogenization, for example, has been associated with drug crystallization and loss of adhesive properties, ultimately reducing transdermal delivery, and dilution effects can modify rheology and skin penetration [21]. Similarly, the type of base and incorporation method can alter stability and efficacy [22]. Topical products also undergo “in-use” metamorphosis (evaporation, crystallization, viscosity drift) affecting release and permeation [23]. Together, these factors rationalize the formulation-dependent uniformity seen here and underscore the need to tailor mixing conditions to API dose, wetting behavior, density mismatch, and vehicle rheology. In the present study, these functional CQAs (e.g., rheology/spreadability/adhesiveness, microstructure, and release/permeation) were not directly measured; thus, the DOE and set-points reported here are optimized with content uniformity as the primary endpoint.

From a mechanistic standpoint, formulation-dependent sensitivity to EMP settings is consistent with established drivers of uniformity in semisolid dosage forms: the physical state of the API (molecularly dissolved versus dispersed as a suspension), particle wetting/agglomeration behavior, density mismatch between dispersed phase and vehicle, and the vehicle’s microstructure and rheology. Regulatory and scientific frameworks for topical products emphasize that process parameters can change microstructure and, consequently, functional performance measures, such as drug release, even when composition remains constant [24,25].

Low-dose, poorly soluble APIs incorporated as suspended solids are generally more sensitive to shear history because adequate mixing energy is required to de-agglomerate particles, improve wetting, and maintain homogeneous dispersion, thereby limiting stratification during and after compounding. This behavior aligns with the literature in which manufacturing variables (including homogenization speed and time) materially influence the viscosity/microstructure and in vitro release behavior of clobetasol propionate creams, illustrating that processing changes can alter semisolid structure and performance [26].

Similarly, petrolatum-based matrices can exhibit strong shear sensitivity because the semicrystalline hydrocarbon network is mechanically disrupted by mixing, which can alter viscosity, thixotropy, and spreadability, and can also improve dispersion of suspended solids such as salicylic acid. Mixing-induced microstructural changes in white petrolatum and salicylic-acid ointment have been shown to modify rheology and can measurably affect drug permeation, underscoring why rpm/time effects may be formulation-specific for ointment-like systems [27].

In contrast, systems in which the API is largely dissolved in the vehicle (or rapidly dissolves during processing) are expected to show reduced sensitivity to shear-driven dispersion effects, because uniformity becomes less dependent on particle break-up and more dependent on ensuring complete dissolution and adequate macro-mixing. This provides a plausible basis for the comparatively modest sensitivity observed in the highly water-soluble urea system within the studied mixing window, where time primarily ensures dissolution while speed mainly influences entrained air and handling properties.

For oxidation- and light-sensitive actives, such as hydroquinone, the compounding process is also relevant to chemical stability because oxygen incorporation, metal contact, and light exposure can accelerate degradation in topical preparations. Published studies on compounded hydroquinone formulations highlight stability vulnerabilities and the influence of formulation choices on delivery and stability; accordingly, minimizing avoidable oxygen/light exposure during preparation is a prudent operational consideration. In the present study, forced degradation was used to support assay specificity rather than to claim product photostability, but these literature considerations contextualize why controlled, closed-jar processing and appropriate packaging are relevant when working with oxidation-prone APIs [28,29].

Forced-degradation results further contextualize process selection. Clobetasol showed pronounced base lability, whereas the diclofenac–hyaluronic acid gel was highly unstable under acidic conditions, aligning with known degradation pathways. Stress testing is a critical element of pharmaceutical development because it reveals intrinsic stability, degradation routes, and potential degradants, thereby underpinning stability-indicating method validation and risk control [30,31]. Within the neutral, ambient, closed-system conditions typical of compounding with the Unguator™, degradation effects are unlikely to confound content-uniformity outcomes; rather, dispersion efficiency is the primary driver of between-strata differences for the low-dose emulsion.

In this study, the DoE evaluated only two process factors (mixing time and rotation speed) because these are the primary, directly programmable EMP parameters governing mixing energy input and are readily standardized across sites. Other potentially relevant variables (jar size/fill level and headspace, blade configuration/orientation, staging of component addition, and initial product temperature) were standardized by protocol to avoid confounding and to maintain feasibility across five formulation matrices; these factors will be prioritized in follow-up studies, starting with fill level/headspace and staged addition.

For clobetasol, the response-surface analysis indicated that rotation speed (rpm) had a stronger influence than mixing time on content uniformity, consistent with the need for higher mixing energy to disrupt agglomerates and reduce stratification in low-dose suspensions. For urea, rpm also predominated, although all conditions remained close to the target assay range. Diclofenac similarly showed a stronger dependence on rpm than time, while the urea–salicylic acid system exhibited formulation-dependent behavior: the urea assay showed only modest sensitivity within the studied range, whereas the salicylic-acid assay was more time-sensitive, consistent with a wetting/dissolution-limited dispersion step under these conditions. These patterns are consistent with mixing theory and published observations that higher rotation speeds shorten the path to blend homogeneity when particle size heterogeneity and wetting limitations are present, albeit with a practical ceiling to avoid over-mixing or air entrapment [32,33,34]. Particle size and solubility further modulate outcomes; smaller particles can improve distribution yet increase cohesive agglomeration at low shear, extending the time to homogeneity [35], and differences in dissolution kinetics across particle sizes can translate to altered bioavailability and distribution [36]. In practice, this means that low-dose, poorly wetting, or density-mismatched APIs profit from higher shear and adequate time, whereas polymer-rich gels may require capped rpm with sufficient duration to balance shear against bubble formation.

Translating model optima to device set-points demonstrated good concordance despite discrete speed/time increments in the Unguator™ control scheme. As summarized in Table 8, F1 mapped exactly (600 rpm/2 min). F2 required a modest reduction from the modeled 1860 rpm to the practical 1650 rpm at 10 min without loss of uniformity. F3 favored a longer time at slightly lower rpm (800 rpm/10 min) relative to the modeled 960 rpm/2.8 min, consistent with curvature observed in viscous gels. F4 supported two windows (mid-rpm/long-time or higher-rpm/shorter-time) both of which were closely approximated by the available device levels (1400 rpm/10 min; 1900 rpm/7:15 min). F5 remained robust across the mid-high region, allowing a small downward rpm adjustment. Confirmation runs at these practicable set-points met the prespecified uniformity criteria, indicating that rational trade-offs between time and speed can reproduce response-surface performance within routine device constraints.

Operationally, both pre-programmed cycles (“Emulsion,” “Emulsion+”) and customized parameter sets achieved reproducible outcomes. The former reduces operator variability and streamlines routine work; the latter permits adaptation to challenging systems where dose, particle size distribution, or excipient interactions demand targeted shear-time combinations. Coupled with the closed, disposable-jar workflow, these features support GMP-aligned hygienic control and minimize cross-contamination risk, as corroborated by the cleaning validation where post-sanitization residues were below detection and well under the 10-ppm acceptance limit [5,16].

QbD is typically implemented as a lifecycle program; however, in compounding practice, the most operationalizable elements are explicit definition of target attributes, identification of the process parameters that drive those attributes, and establishment of actionable operating ranges and controls. In this study, we, therefore, focused on: (i) QTPP and CQA definition for semisolid compounding, (ii) experimental evaluation of CPPs (rpm and time) via DOE against the primary CQA (content uniformity), (iii) definition of an operational design space bounded to the studied rpm/time ranges (Table 9), and (iv) verification of hygiene controls via cleaning/cross-contamination validation. This condensed risk-mapping approach is aligned with the tool-based risk assessment concepts described in ICH Q9(R1) and is presented here in a pragmatic format suitable for routine compounding implementation [8].

To make the CPP/CMA–CQA linkage and risk assessment explicit, Table 9 provides a condensed FMEA-style mapping and risk ranking, together with controls informed by the present data. Based on this mapping, a practical control strategy for routine EMP compounding is: (a) standardize input handling (weighing accuracy, incorporation order, and jar fill volume/headspace) and record device set-points batch-to-batch; (b) control CPPs using the formulation-appropriate operational windows in Table 8 (e.g., high energy/adequate time for low-dose suspensions; capped rpm with sufficient time for polymeric gels to minimize air entrapment); (c) apply pre-validated device programs (Emulsion/Emulsion+) when multi-phase or thermally influenced steps are required; and (d) maintain hygiene controls through the validated cleaning/sanitization procedure and periodic verification against the acceptance limit. This control strategy should be viewed as a step toward broader lifecycle QbD implementation; additional work (e.g., multi-operator/multi-site robustness, broader CMA variation, and expanded CQAs, such as rheology and release) is needed for comprehensive lifecycle validation.

From an implementation perspective, for several formulations (e.g., F1) the device permitted exact implementation of the predicted set-point, whereas for other formulations, minor parameter rounding to the nearest available rpm/time step or programmatic adjustments were required due to equipment granularity and predefined mixing levels. Importantly, confirmation batches compounded at the practical set-points met the predefined homogeneity criteria (90–110% label claim; CV ≤ 5%), indicating that modest deviations from the model optima did not compromise content uniformity.

It is also important to mention that this study has limitations. First, all APIs, semisolid vehicles (Versatile™ and Nourivan Antiox™), and the EMP device/jar/blade system were sourced from a single manufacturer. Second, while the selected formulations are representative in terms of physicochemical challenge (high solids, low-dose suspension, polymeric gel, multicomponent system, oxidation-sensitive API), they do not encompass the full range of semisolid microstructures used in practice, and the DOE levels reflect a practical operating space rather than an exhaustive exploration of shear history or temperature effects. A further limitation is that impurity formation in the compounded products was not comprehensively profiled (e.g., identification/qualification of degradants or impurity mass balance) beyond the forced-degradation work used to demonstrate assay specificity. Although the validated UHPLC methods were designed to be stability-indicating and closed-jar processing was used to limit avoidable oxygen/light exposure, oxidation- or light-sensitive APIs (e.g., hydroquinone) warrant targeted impurity monitoring and formal photostability/stability studies in future work if longer BUDs or product-specific stability claims are intended. Future work should also include broader base classes (including anhydrous ointments and other gel networks), rheometry and air-content quantification, and particle-size/shape analytics. Additionally, the DOE-driven optimization and the reported operating windows were defined using assay-based content uniformity outcomes (mean % label claim and strata variability). Importantly, the present study did not include a head-to-head comparison with conventional manual mortar-and-pestle compounding or with other automated mixing devices used in routine practice. Comparative studies are warranted to benchmark homogeneity, operator-to-operator variability, and process robustness against manual and alternative devices. Finally, a pilot microplastics screen (single determinations with FTIR-based polymer identification) suggested polyethylene particles in petrolatum-based materials; this observation is hypothesis-generating and was not used for quantitative risk assessment

From a transferability perspective, the qualitative trends observed here are expected to hold across many semisolid systems, even if the quantitative rpm/time values do not. Specifically, low-dose APIs and poorly wetting or density-mismatched powders generally require higher specific mixing energy and/or longer effective mixing time to avoid agglomeration and stratification, whereas structured polymeric gels may benefit from capped rpm with longer duration to balance dispersion against air entrapment. Multicomponent systems may exhibit more than one viable operating window depending on which component is rate-limiting for wetting and dispersion. However, the numeric set-points reported in this study are device- and base-dependent because shear field, flow pattern, and energy dissipation vary with mixer geometry, blade design, jar dimensions, fill volume/headspace, and vehicle rheology.

Nevertheless, the combined validation, degradation, homogeneity, and DOE findings provide a coherent, QbD-aligned step toward model-informed, device-encoded recipes that can improve parameter control and reproducibility while respecting formulation-specific constraints, reinforcing the Unguator™ as a versatile platform for semisolid preparation in compounding practice.

## 5. Conclusions

This work demonstrates that the Unguator™ (EMP) enables standardized, reproducible compounding of semisolid pharmaceutical preparations with respect to assay-based content uniformity. Validated, stability-indicating UHPLC methods confirmed assay suitability and supported interpretation of content uniformity outcomes. Across chemically and rheologically diverse formulations, the device achieved layer-to-layer content uniformity within predefined criteria, with low-dose suspensions and multicomponent systems showing the greatest sensitivity to process energy and time—findings clarified by the DOE response-surface analysis. Comparative benchmarking versus manual mortar-and-pestle preparation and other mixers remains to be established.

Translation of model-predicted optima into practicable device set-points (within the EMP’s discrete rpm/time increments and pre-validated programs) maintained target uniformity, indicating that modest, rational trade-offs between speed and duration can reproduce optimal dispersion. Cleaning validation using hydroquinone as a tracer showed post-sanitization residues below the LOQ and well under the acceptance limit, supporting minimal cross-contamination risk in a closed-system workflow.

Taken together, these results support the Unguator™ as a robust and adaptable platform for professionalized compounding: pre-programmed cycles (“Emulsion,” “Emulsion+”) reduce operator variability for routine preparations, while parameter customization enables formulation-specific control of critical quality attributes in more challenging matrices. Within the study’s practical operating space, the approach aligns with QbD principles and GMP-consistent hygiene control.

## Figures and Tables

**Table 1:** Model formulations compounded using the Unguator™ system.

Formulation Code	Composition	Vehicle	Formulation Type	Pharmaceutical Intent
F1	Urea 40%	Versatile™	Cream	High-concentration keratolytic [12]
F2	Clobetasol propionate 0.05%	Versatile™	Cream	Low-dose corticosteroid for inflammatory dermatoses [13]
F3 *	Diclofenac sodium 3.0% + Hyaluronic acid 2.5%	In-house gel base *	Gel	Polymeric high-viscosity system [14]
F4	Urea 10% + Salicylic acid 1%	Versatile™	Cream	Multicomponent keratolytic [12]
F5	Hydroquinone 5%	Nourivan Antiox™	Cream	Oxidation-sensitive depigmenting agent [15]

* F3 was prepared via a two-step process (gel base preparation followed by API incorporation).

**Table 2:** Summary of UHPLC method conditions used for API quantification and validation.

Formulation	Mobile Phase Composition	Work Concentration (mg/mL)	Diluent Solution	Injection Volume (µL)	Column	Flow Rate (mL/min)	Detection Wavelength (nm)
F1	Solution A (0.001% formic acid): Solution B (Acetonitrile) (5:95, *v*/*v*), gradient mode *	0.40	Acetonitrile:Water (90:10, *v*/*v*)	10.0	OH5 150 × 4.6 mm (01) ^1^	1.0	195
F2	Phosphate buffer 0.0083 M:Acetonitrile:Methanol (85:95:25, *v*/*v*/*v*)	0.02	Methanol	1.0	C18 50 × 2.1 mm (04) ^2^	0.4	240
F3	Methanol:Phosphate buffer (70:30, *v*/*v*)	0.30	Methanol:Water (70:30, *v*/*v*)	0.1	C08 50 × 2.1 mm (U-03) ^3^	0.3	274
F4—Urea	Solution A (0.001% formic acid):Solution B (Acetonitrile) (5:95, *v*/*v*), gradient mode *	0.02	Acetonitrile:Water (90:10, *v*/*v*)	10.0	OH5 150 × 4.6 mm (01) ^1^	1.0	195
F4–Salicylic acid	Methanol:Glacial Acetic Acid:Water (40:1:60, *v*/*v*/*v*)	0.04	Acetonitrile:Water (90:10, *v*/*v*)	10.0	C08 150 × 4.6 mm (06) ^4^	1.5	240
F5	Methanol:Water (55:45, *v*/*v*)	0.25	Mobile Phase	0.5	C18 100 × 2.1 mm (U-01) ^5^	0.1	280

* Gradient mode was applied by setting Channel A (5% formic acid solution) and Channel B (95% acetonitrile) at a flow rate of 1.0 mL/min. Column stabilization was performed for 30 min prior to sample injection to ensure baseline consistency and optimal separation. ^1^ Ascentis^®^ Express OH5 (USP L86), C18, 150 × 4.6 mm, 2.7 µm—Millipore Sigma, Darmstadt, Germany. ^2^ Inertsil ODS (USP L1), C18, 50 × 2.1 mm, 5 µm–GL Sciences Inc., Tokyo, Japan. ^3^ Hypersil GOLD C8 (USP L3), 50 × 2.1 mm, 3 µm—Thermo Fisher Scientific, Waltham, MA, USA. ^4^ Inertsil C8 (USP L3), 150 × 4.6 mm, 5 µm—GL Sciences Inc., Tokyo, Japan. ^5^ Hypersil GOLD C18 (USP L1), 100 × 2.1 mm, 3 µm—Thermo Fisher Scientific, Waltham, MA, USA.

**Table 3:** 3^2^ factorial design to determine optimal homogenization conditions in nine replications.

Issue	X_1_	X_2_	X_1_^2^	X_2_^2^	X_1_X_2_	Responses (*n* = 9)
1	−1	−1	1	1	1	Y1
2	−1	0	1	0	0	Y2
3	−1	1	1	1	−1	Y3
4	0	−1	0	1	0	Y4
5	0	0	0	0	0	Y5
6	0	1	0	1	0	Y6
7	1	−1	1	1	−1	Y7
8	1	0	1	0	0	Y8
9	1	1	1	1	1	Y9

X_1_ (Mixing time, min): (−1) = 2; (0) = 6; (1) = 10; X_2_ (Rotation speed, rpm): (−1) = 600; (0) = 1500; (1) = 2400.

**Table 4:** Summary of validation results of the ultra-high-performance liquid chromatographic methods.

	Linearity				Limits and Noise		Specificity	Precision		Accuracy
Formulation	Range (µg/mL)	Analytical Curve	r	ANOVA’s Significance of Regression (F)	LOD (µg/mL)	LOQ (µg/mL)	Discrepancy (%)	Repeatability (CV, %)	Intermediate Precision (CV, %)	Recovery (%)
Acceptance criteria	-	-	≥0.99	Significant regression (*p* < 0.05; F_reg_ > F_crit_)	Reported (fit-for-purpose)	LOQ ≤ lowest calibration level used for assay	<2.0	≤5.0	≤5.0	100 ± 2
F1	285.04 − 529.36	y = 0.0418x − 0.4547	0.9997	19,217.44	2.52	7.23	1.49 (6.08)	3.47	3.77	99.60 (0.42)
F2	14.56 − 27.04	y = 0.0845x − 0.044	0.9961	1644.34	0.0173	0.052	1.94 (5.38)	4.64	4.97	99.74 (2.41)
F3	70.70 − 131.30	y = 0.011x − 0.0167	0.9972	2353.35	0.18	0.53	0.47 (5.29)	4.14	3.99	99.63 (0.87)
F4–Urea	285.04 − 529.36	y = 0.0418x − 0.4547	0.9997	19,217.44	2.52	7.23	1.49 (6.08)	3.47	3.77	99.60 (0.42)
F4–Salicylic acid	28.42 − 52.78	y = 0.0764x − 0.1399	0.9984	3984.31	0.023	0.069	0.25 (1.13)	2.23	1.93	100.54 (1.49)
F5	177.80 − 330.20	y = 0.0764x − 0.1399	0.9953	1386.81	0.04	0.13	1.94 (0.95)	4.29	2.46	98.47 (1.70)

CV = Coefficient of Variation; LOD = Limit of Detection; LOQ = Limit of Quantification (20-μL injections). Where applicable, results are reported as mean (standard deviation, SD) (SD shown in parentheses for discrepancy and recovery), while precision is expressed as CV (%); linearity (r), regression (F), and LOD/LOQ are model-derived parameters for which SD is not applicable.

**Table 5:** Forced-degradation outcomes supporting specificity of the UHPLC assay.

Formulation	HCl * (%d)	Peak-Purity (P/F)	NaOH * (%d)	Peak-Purity	Photolysis/UV * (%d)	Peak-Purity	Heat * (%d)	Peak-Purity	H_2_O_2_ * (%d)	Peak-Purity
F1	−12.30 (0.58)	P	−26.20 (0.32)	P	−11.38 (0.32)	P	−2.53 (3.99)	P	−5.28 (0.61)	P
F2	−9.03 (1.20)	P	−49.37 (0.04)	F	−13.17 (1.04)	P	8.89 (1.19)	P	1.12 (1.07)	P
F3	−86.07 (0.56)	P	5.24 (4.07)	P	1.27 (1.74)	P	3.95 (1.94)	P	5.65 (1.57)	P
F4—Urea	−12.30 (0.58)	P	−26.20 (0.32)	P	−11.38 (0.32)	P	−2.53 (3.99)	P	−5.28 (0.61)	P
F4—Salicylic acid	−7.49 (1.25)	P	−32.88 (0.24)	P	1.22 (0.42)	P	−15.24 (0.30)	P	−0.96 (0.74)	P
F5	−3.59 (2.47)	P	−8.99 (6.63)	F	−0.52 (1.93)	P	−2.53 (3.99)	P	−4.57 (5.18)	F

* %d = percentage discrepancy between the API peak area of the stressed sample and the unstressed control; %d < 2% indicates non-significant degradation. Photolysis (light stress) corresponds to the non-ICH Q1B qualitative light-exposure forced-degradation condition described in Section 2 (Specificity), evaluated relative to an unstressed control not submitted to photolysis. Peak purity (P/F) summarizes PDA peak-purity evaluation (Chromeleon™ PPA): Pass indicates peak-purity match factor ≥ 950; Flag indicates < 950. Values for %d are reported as mean (SD), based on replicate analyses (*n* = 6) performed for each stress condition. Numeric peak-purity match-factor outputs are provided in Supplementary Table S1.

**Table 6:** Stratified content uniformity (% label claim) by jar layer (top/middle/bottom) for EMP-compounded formulations (UHPLC).

Formulation	Average (%)	SD	CV (%)
F1	96.46	2.20	2.29
F1—Emulsion	96.49	0.99	1.03
F1—Emulsion+	97.78	0.54	0.55
F2	100.09	3.70	3.69
F2—Emulsion	96.23	4.26	4.43
F2—Emulsion+	101.94	4.88	4.79
F3	100.25	2.37	2.37
F3—Emulsion	100.50	2.25	2.24
F3—Emulsion+	97.36	1.66	1.71
F4 (Urea)	99.61	0.95	0.95
F4 (Urea)—Emulsion	97.21	1.64	1.69
F4 (Urea)—Emulsion+	99.51	1.46	1.47
F4 (Salicylic acid)	99.43	2.98	3.00
F4 (Salicylic acid)—Emulsion	108.81	2.63	2.42
F4 (Salicylic acid)—Emulsion+	99.61	2.07	2.08
F5	100.24	3.69	3.68
F5—Emulsion	99.98	0.62	0.62
F5—Emulsion+	101.32	1.24	1.22

Results are expressed relative to the label content for each API. SD (standard deviation) and CV (%) (coefficient of variation) reflect dispersion across the three stratified jar layers (top, middle, bottom; *n* = 3) for each condition.

**Table 7:** Model statistics and predicted optima from response-surface analysis (content uniformity, assay).

Formulation	Issue	Results (Responses: % Label Claim)									
		y1	y2	y3	y4	y5	y6	y7	y8	y9	y10
F1	1	94.65	94.63	93.72	99.48	99.43	99.50	95.55	95.65	95.50	96.46
	2	92.46	92.98	92.70	91.41	91.58	91.95	95.83	95.88	95.98	93.42
	3	91.70	91.48	91.52	97.68	96.97	97.18	90.80	91.11	91.10	93.28
	4	94.30	94.64	94.77	94.86	94.99	94.96	95.07	95.60	95.38	94.95
	5	92.22	92.49	92.69	92.97	92.89	92.97	95.43	95.41	96.13	93.69
	6	99.72	100.12	100.09	92.90	93.23	93.16	92.74	92.20	92.74	95.21
	7	92.99	93.22	93.11	98.03	98.49	98.29	95.08	95.23	95.52	95.55
	8	92.79	92.85	92.35	92.77	93.18	92.91	94.75	94.77	94.80	93.46
	9	91.80	91.59	92.09	93.73	93.79	94.12	97.78	97.78	97.64	94.48
F2	1	106.47	106.04	106.92	107.48	107.64	107.40	111.85	112.03	112.00	108.65
	2	104.49	104.50	104.48	103.45	103.68	103.42	98.30	98.43	98.54	102.14
	3	85.74	85.62	85.62	83.20	83.21	83.18	85.28	85.40	85.46	84.75
	4	90.75	90.83	90.90	82.33	82.45	82.41	86.02	86.24	85.78	86.41
	5	84.20	84.03	84.00	84.80	85.01	84.73	78.38	78.28	78.28	82.41
	6	113.51	113.45	113.51	102.37	102.43	102.43	109.95	110.92	110.92	108.83
	7	117.26	117.74	117.74	121.55	121.62	121.55	114.92	114.49	114.55	117.94
	8	97.49	97.18	97.24	97.66	97.60	97.72	105.40	105.21	105.34	100.09
	9	91.52	91.46	91.52	98.56	98.56	99.05	102.97	103.28	103.40	97.81
F3	1	104.73	102.60	102.41	101.02	100.66	100.93	100.35	107.09	103.45	102.58
	2	94.37	95.58	94.07	99.22	102.86	101.12	98.86	98.82	103.56	98.72
	3	99.44	101.89	101.01	98.32	98.64	98.91	98.67	98.69	98.74	99.37
	4	97.88	99.41	100.49	102.61	103.87	103.47	96.99	98.55	98.96	100.25
	5	97.37	96.32	96.80	99.21	103.34	104.28	103.50	104.72	105.22	101.20
	6	92.57	92.29	99.56	90.53	93.02	93.56	88.04	90.71	91.52	92.42
	7	105.04	105.04	105.51	103.95	104.21	103.52	97.64	97.68	100.55	102.57
	8	84.81	84.81	85.72	89.96	84.86	86.97	86.46	89.78	89.06	86.94
	9	98.50	99.38	98.38	94.55	97.14	97.50	98.44	99.78	98.94	98.07
F4 (Urea)	1	99.39	99.02	98.78	98.23	98.37	98.98	97.84	97.72	98.36	98.52
	2	101.08	100.91	100.97	100.05	100.31	100.61	109.90	110.04	109.99	103.76
	3	101.58	101.79	101.34	99.47	99.96	100.00	103.24	102.93	103.09	101.49
	4	123.53	123.21	123.54	95.43	95.61	95.15	95.20	95.33	95.02	104.67
	5	97.35	97.90	98.22	100.00	99.42	99.67	101.18	101.74	101.02	99.61
	6	99.94	100.11	99.81	98.05	98.20	98.87	99.99	100.79	100.69	99.61
	7	100.37	100.61	100.39	105.50	105.25	105.94	95.57	95.94	96.23	100.64
	8	99.10	98.75	99.26	100.24	99.54	100.25	98.42	99.35	98.85	99.31
	9	101.30	101.17	101.68	101.63	101.77	101.28	102.56	101.78	102.69	101.76
F4 (Salicylic acid)	1	100.78	100.76	100.69	104.27	104.78	104.86	101.73	101.37	101.41	102.29
	2	125.93	125.44	125.98	124.33	124.64	124.25	127.69	128.22	128.26	126.08
	3	113.13	112.96	113.06	107.03	107.08	107.11	106.52	106.56	106.57	108.89
	4	99.12	99.21	98.77	99.50	99.43	99.49	99.28	99.32	99.28	99.27
	5	102.74	102.73	102.71	95.53	95.53	95.47	100.04	100.17	99.91	99.43
	6	105.46	105.92	105.75	102.74	99.89	99.74	97.42	97.06	97.08	101.23
	7	97.57	97.42	97.28	98.55	98.53	98.54	94.81	94.64	94.85	96.91
	8	92.53	92.24	92.59	100.28	100.52	100.76	101.47	101.63	101.77	98.20
	9	101.20	101.37	101.42	102.80	102.73	102.91	103.66	103.68	105.06	102.76
F5	1	100.50	100.47	100.29	106.53	106.57	107.55	106.37	105.52	106.00	107.04
	2	103.01	102.88	103.04	105.23	104.27	104.71	104.01	104.60	104.97	109.37
	3	102.97	103.26	103.29	103.39	103.27	103.11	101.29	101.31	100.79	121.63
	4	101.82	102.57	102.62	102.40	102.99	102.90	100.81	100.91	101.15	124.68
	5	99.64	100.03	99.98	100.76	100.82	101.07	103.71	103.54	103.41	130.06
	6	103.14	102.81	103.25	103.77	104.13	104.66	104.90	103.87	104.29	121.00
	7	101.75	101.59	101.53	100.55	100.93	101.07	104.80	105.31	105.58	121.18
	8	98.36	99.21	99.44	97.73	98.58	98.35	98.91	99.17	99.08	103.08
	9	99.95	100.02	99.62	95.97	96.01	95.77	104.60	105.04	105.14	128.11

Values shown are individual assay results for confirmation batches compounded at the practical set-points; no replicate batches were prepared per identical condition; therefore, mean and SD are not applicable.

**Table 8:** Comparison of response surface optimal conditions versus practical settings for each formulation, and operational design space (within studied ranges) meeting homogeneity criteria (90–110% label claim; strata CV ≤ 5%).

Formulation	Primary Mixing Risk (Context)	Operational Design Space Window Within Studied Factors	Response Surface Optimum	Implementable Practical Set-Point(s) Used/Confirmed
F1 (Urea 40%)	High solids; dispersion robustness	Broadly compliant across studied range (600–2400 rpm; 2–10 min)	600 rpm/2.0 min	600 rpm/2.0 min
F2 (Clobetasol 0.05%)	Low-dose; poorly wetting/agglomeration risk	Higher energy required: ≥1500 rpm with ≥6–10 min (best performance at high rpm/long time)	1860 rpm/10.0 min	1650 rpm/10.0 min (or Emulsion/Emulsion+)
F3 (Diclofenac in HA gel)	Polymeric gel; air entrapment at high rpm	Moderate rpm favored: ~800–1900 rpm with ≥6–10 min; avoid very high rpm	960 rpm/2.8 min	800 rpm/10.0 min
F4 (Urea 10% + Salicylic acid 1%)	Multicomponent; competing optimization windows	Two viable windows: mid-rpm/long-time or higher-rpm/shorter-time within studied range	1500 rpm/10.0 min or 2200 rpm/6.8 min	1400 rpm/10.0 min or 2100 rpm/7.0 min
F5 (Hydroquinone 5%)	Oxidation-sensitive API; robustness	Robust across mid-high region within studied range; mid settings sufficient	1500 rpm/10.0 min	1450 rpm/10.0 min

**Table 9:** Condensed CPP/CMA–CQA mapping and risk ranking (FMEA-style) with controls applied in this study.

CQA	Key Failure Mode/Risk Driver	Candidate CMAs (Examples)	CPPs (Studied vs. Identified)	Risk (Pre-Control)	Controls/Mitigations Implemented in This Study	Residual Gap (Future Work)
Content uniformity (primary)	Stratification; incomplete wetting; agglomeration	API particle size/wettability; density mismatch; solids loading; vehicle rheology	Studied: rpm, time (DOE). Identified: incorporation order, jar fill volume/headspace, scraping/handling, program selection	High	DOE + confirmation runs; stratified sampling with acceptance 90–110% and CV ≤ 5%; operational design space (Table 9); implementable set-points (Table 8)	Robustness across wider CMA ranges; multi-operator/multi-site verification
Chemical stability (supporting)	Oxidation/degradation during/after compounding	API intrinsic stability; antioxidant capacity; packaging/light/oxygen exposure	Identified: exposure time; thermal program selection	Medium	Stability-indicating UHPLC; forced degradation to confirm specificity; use of antioxidant vehicle for HQ; opaque packaging	Real-time stability/BUD under broader storage conditions
Cross-contamination/residues (hygiene)	Carry-over between batches	API adherence/solubility; surface interactions	CPP: cleaning/sanitization procedure	Medium–High	Cleaning validation with HQ tracer; residues below LOQ and <10 ppm acceptance; closed, disposable-jar workflow	Broader API panel and worst-case carry-over evaluation
Physical acceptability (secondary)	Excess air entrapment; poor spreadability	Vehicle rheology; polymer content	Identified: rpm ceiling for gels; time balance	Medium	DOE-informed recommendation to cap rpm for polymeric gels; visual inspection for gross defects	Quantitative rheology/air content; in-use metamorphosis studies

## Data Availability

The data presented in this study are available within the article. Additional raw datasets supporting the conclusions—including chromatographic output files, calibration regression outputs (slope/intercept/R^2^ and residual plots), forced-degradation chromatograms, and Microsoft Excel DoE files (ANOVA tables, regression statistics including R^2^, fitted equations, and diagnostic plots)—are available from the corresponding author upon reasonable request.

## Supplementary Materials

The following supporting information can be downloaded at: https://www.mdpi.com/article/10.3390/pharmaceutics18020205/s1, Table S1: PDA peak-purity match-factor outputs (Chromeleon™ PPA) for forced-degradation samples.

## References

[1] Campbell E.H., Elston D.M., Straughan C.L. (2020). A Review of the Clinical Indications, General Principles and Techniques Related to Compounding. J. Am. Acad. Dermatol..

[2] Chauhan L., Gupta S. (2020). Creams: A Review on Classification, Preparation Methods, Evaluation and Its Applications. J. Drug Deliv. Ther..

[3] Ferreira A.D.O. (2023). Guia Prático Da Farmácia Magistral.

[4] Leong T.S.H., Wooster T.J., Kentish S.E., Ashokkumar M. (2009). Minimising Oil Droplet Size Using Ultrasonic Emulsification. Ultrason. Sonochem..

[5] United States Pharmacopeia (2024). USP General Chapter <795>: Pharmaceutical Compounding—Nonsterile Preparations.

[6] Piette M., Stassen T., Kinget R., Delattre L. (2006). Validation Study of the Unguator, an Apparatus for Compounding Dermatological Preparations. Int. J. Pharm. Compd..

[7] FagronLabTM EMP Electronic Mortar and Pestle Technology. http://www.fagronlab.com.

[8] International Council for Harmonisation of Technical Requirements for Pharmaceuticals for Human Use (ICH) (2023). ICH Q9(R1): Quality Risk Management.

[9] International Council for Harmonisation of Technical Requirements for Pharmaceuticals for Human Use (ICH) (2009). ICH Q8(R2): Pharmaceutical Development.

[10] International Council for Harmonisation of Technical Requirements for Pharmaceuticals for Human Use (ICH) (2008). ICH Q10: Pharmaceutical Quality System.

[11] Winnicki A.M., Partyka D., Jachowicz K., Macherowski T., Marchwiński Ł., Nȩdza P., Stebnicka M., Sulejewska J. (2013). Dimethicone Impact on Aeration of Suppository Mass in Unguator Mixing Machine. Curr. Issues Pharm. Med. Sci..

[12] Celleno L. (2018). Topical Urea in Skincare: A Review. Dermatol. Ther..

[13] Feldman S.R., Yentzer B.A. (2009). Topical Clobetasol Propionate in the Treatment of Psoriasis. Am. J. Clin. Dermatol..

[14] Kuzmina M.V., Shlyk I.V., Panafidina V.A., Kozhevin A.A., Polushin Y.S., Krivov V.O. (2023). The Use of a Combination of Diclofenac and Orphenadrine for Analgesia in Knee Replacement. Messenger Anesthesiol. Resusc..

[15] Fabian I.M., Sinnathamby E.S., Flanagan C.J., Lindberg A., Tynes B., Kelkar R.A., Varrassi G., Ahmadzadeh S., Shekoohi S., Kaye A.D. (2023). Topical Hydroquinone for Hyperpigmentation: A Narrative Review. Cureus.

[16] U.S. Food and Drug Administration (2024). ICH Harmonised Guideline—Validation of Analytical Procedures Q2(R2).

[17] ORA Laboratory (2019). Manual, Volume II: Ensuring the Quality of Test Results (ORA-LAB.5.9) 2019.

[18] Cantwell H. (2025). Eurachem Guide: The Fitness for Purpose of Analytical Methods—A Laboratory Guide to Method Validation and Related Topics.

[19] ORA Laboratory (2023). Manual, Volume II: Methods, Method Verification and Validation (ORA-LAB.5.4.5) 2023.

[20] United States Pharmacopeial Convention USP (2021). United States Pharmacopeial Convention USP <1225> Validation of Compendial Procedures 2021.

[21] Pharmaceutical Inspection Co-operation Scheme (PIC/S) (2007). Recommendation on Validation Master Plan, Installation and Operational Qualification, Non-Sterile Process Validation and Cleaning Validation 2007.

[22] European Medicines Agency (2014). Guideline on Setting Health Based Exposure Limits for Use in Risk Identification in the Manufacture of Different Medicinal Products in Shared Facilities 2014.

[23] World Health Organization (2019). Cleaning Validation. WHO Expert Committee on Specifications for Pharmaceutical Preparations. Fifty-Third Report.

[24] Mohamed L.A., Kamal N., Elfakhri K.H., Willett D., Wokovich A., Strasinger C., Cruz C.N., Raney S.G., Ashraf M., Zidan A.S. (2020). Drug Recrystallization in Drug-in-Adhesive Transdermal Delivery System: A Case Study of Deteriorating the Mechanical and Rheological Characteristics of Testosterone TDS. Int. J. Pharm..

[25] Nagelreiter C., Kratochvilova E., Valenta C. (2015). Dilution of Semi-Solid Creams: Influence of Various Production Parameters on Rheological Properties and Skin Penetration. Int. J. Pharm..

[26] Jin X., Imran M., Mohammed Y. (2022). Topical Semisolid Products—Understanding the Impact of Metamorphosis on Skin Penetration and Physicochemical Properties. Pharmaceutics.

[27] European Medicines Agency (2018). Guideline on Quality and Equivalence of Locally Applied, Locally Acting Cutaneous Products 2018.

[28] Shah V.P. (2022). In Vitro Release Test (IVRT): Principles and Applications. Int. J. Pharm..

[29] Fauzee A.F.B., Khamanga S.M., Walker R.B. (2014). The Impact of Manufacturing Variables on in Vitro Release of Clobetasol 17-Propionate from Pilot Scale Cream Formulations. Drug Dev. Ind. Pharm..

[30] Kitagawa S., Fujiwara M., Okinaka Y., Yutani R., Teraoka R. (2015). Effects of Mixing Procedure Itself on the Structure, Viscosity, and Spreadability of White Petrolatum and Salicylic Acid Ointment and the Skin Permeation of Salicylic Acid. Chem. Pharm. Bull..

[31] Matsubayashi T., Sakaeda T., Kita T., Nakamura T., Kakumoto M., Funasaka Y., Ichihashi M., Fujita T., Kamiyama F., Yamamoto A. (2003). Effects of Various Storage Conditions and Alterations of Antioxidant Contents on Chromatic Aberration of Hydroquinone Ointment. Biol. Pharm. Bull..

[32] Serrano D.R., Gordo M.J., Matji A., González S., Lalatsa A., Torrado J.J. (2019). Tuning the Transdermal Delivery of Hydroquinone upon Formulation with Novel Permeation Enhancers. Pharmaceutics.

[33] Roberto de Alvarenga Junior B., Lajarim Carneiro R. (2019). Chemometrics Approaches in Forced Degradation Studies of Pharmaceutical Drugs. Molecules.

[34] Bonciarelli S., Desantis J., Goracci L., Siragusa L., Zamora I., Ortega-Carrasco E. (2021). Automatic Identification of Lansoprazole Degradants under Stress Conditions by LC-HRMS with MassChemSite and WebChembase. J. Chem. Inf. Model..

[35] Alyami H., Dahmash E., Bowen J., Mohammed A.R. (2017). An Investigation into the Effects of Excipient Particle Size, Blending Techniques & Processing Parameters on the Homogeneity & Content Uniformity of a Blend Containing Low-Dose Model Drug. PLoS ONE.

[36] Lee W.B., Widjaja E., Heng P.W.S., Chan L.W. (2020). The Effect of Rotation Speed and Particle Size Distribution Variability on Mixability: An Avalanche Rheological and Multivariate Image Analytical Approach. Int. J. Pharm..

