# Supplementary material for: Quality-by-Design Compounding of Semisolids Using an Electronic Mortar and Pestle Device for Compounding Pharmacies: Uniformity, Stability, and Cleaning
Source: Pharmaceutics. 2026 Feb 4;18(2):205. doi: 10.3390/pharmaceutics18020205 (PMC12944681; doi:10.3390/pharmaceutics18020205)
Supplement: Supplementary file 1 [file pharmaceutics-18-00205-s001.zip › pharmaceutics-4026438-supplementary.pdf]

Supplementary Materials

Table S1. PDA peak-purity match-factor outputs (Chromeleon™ PPA) for forced-degradation samples.

Formulation	HCl *(%d)	Peak-purity	NaOH *(%d)	Peak-purity	UV *(%d)	Peak-purity	Heat *(%d)	Peak-purity	H_2O_2 *(%d)	Peak-purity
F1	-12.3	998	-26.2	1000	-11.38	1000	-2.53	1000	-5.28	1000
F2	-9.03	997	-49.37	520	-13.17	993	8.89	995	1.12	998
F3	-86.07	989	5.24	999	1.27	999	3.95	1000	5.65	1000
F4 – Urea	-12.3	998	-26.2	1000	-11.38	1000	-2.53	1000	-5.28	1000
F4 – Salicylic acid	-7.49	996	-32.88	967	1.22	997	-15.24	996	-0.96	994
F5	-3.59	987	-8.99	670	-0.52	985	-2.53	985	-4.57	887

Peak purity values correspond to the Chromeleon™ Peak Purity Analysis (PPA) match factor (0–1000; ideal ≈ 1000). * Values are reported as the mean of replicate analyses for each stress condition.
